# Neural Representations of Airflow in *Drosophila* Mushroom Body

**DOI:** 10.1371/journal.pone.0004063

**Published:** 2008-12-30

**Authors:** Akira Mamiya, Jennifer Beshel, Chunsu Xu, Yi Zhong

**Affiliations:** 1 Cold Spring Harbor Laboratory, Cold Spring Harbor, New York, United States of America; 2 SUNY Stony Brook, Stony Brook, New York, United States of America; University of Maryland, United States of America

## Abstract

The *Drosophila* mushroom body (MB) is a higher olfactory center where olfactory and other sensory information are thought to be associated. However, how MB neurons of *Drosophila* respond to sensory stimuli other than odor is not known. Here, we characterized the responses of MB neurons to a change in airflow, a stimulus associated with odor perception. *In vivo* calcium imaging from MB neurons revealed surprisingly strong and dynamic responses to an airflow stimulus. This response was dependent on the movement of the 3^rd^ antennal segment, suggesting that Johnston's organ may be detecting the airflow. The calyx, the input region of the MB, responded homogeneously to airflow on. However, in the output lobes of the MB, different types of MB neurons responded with different patterns of activity to airflow on and off. Furthermore, detailed spatial analysis of the responses revealed that even within a lobe that is composed of a single type of MB neuron, there are subdivisions that respond differently to airflow on and off. These subdivisions within a single lobe were organized in a stereotypic manner across flies. For the first time, we show that changes in airflow affect MB neurons significantly and these effects are spatially organized into divisions smaller than previously defined MB neuron types.

## Introduction

The integration of information from multiple sensory modalities is a fundamental feature of neural processing [1]. Many higher associational areas of the brain obtain sensory information from diverse sources and integrate these inputs to produce a coherent representation of the world [2], [3], [4], [5], [6]. The mushroom body (MB) of insects is one such brain region [7]. In most insects, it is considered to be a higher olfactory center receiving its olfactory inputs from antennal lobe projection neurons (PNs), which in turn receive olfactory inputs from olfactory sensory neurons [8]. It is also thought to receive and integrate sensory information from different modalities [8] to form olfactory associative memory [9], [10]. Genetic and behavioral investigations of olfactory associative memory in the *Drosophila* MB have greatly advanced our understanding of the various genetic components required for this process [11]. However, the manner by which the interaction and integration of information garnered from multiple sources occurs in the *Drosophila* MB remains poorly understood. Although extensive efforts have been devoted to studying odor representations in the *Drosophila* MB [12], [13], [14], the only other form of sensory representation reported to date in the *Drosophila* MB is that of electric shock [14], [15]. Here, we characterize for the first time the neural representations of airflow in the *Drosophila* MB.

We chose to study the neural representations of airflow in the MB because of the close relationship between olfactory perception and airflow. Odorant molecules in the environment can be conveyed to an animal's olfactory sensory neurons via wind or water currents[16], and fluctuations in air or water flow across the olfactory organ affects olfactory perception in many species [17], [18], [19], [20], [21], [22], [23]. Despite this close relationship, how air/water flow information is combined with odor information to form olfactory perception is not known. Studying how an airflow stimulus is represented by neurons in the *Drosophila* MB should provide us with valuable clues for understanding olfactory perception. Furthermore, it may give us insights into the neural mechanisms underlying the integration of olfactory and airflow information.

The *Drosophila* MB is composed of approximately 2500 neurons [24]. MB neurons can be divided into three types according to their axonal projection pattern [25]. Axons of α/β neurons bifurcate to form a vertical α lobe and a horizontal β lobe. Axons of α'/ β' neurons also bifurcate to form a vertical α' lobe and a horizontal β' lobe that run parallel to the α and β lobes. Axons of γ neurons do not bifurcate and form a horizontal γ lobe located anterior to the β and β' lobes. Behavioral and imaging studies have suggested that these three types of anatomically- defined neurons also have different functional roles [14], [26], [27], [28], [29], [30], [31]. A recent anatomical study has shown that dendrites of MB neurons can be segregated into 17 complementary domains according to their neuroblast clonal origins and birth orders, suggesting that each type of MB neuron may be composed of different anatomical subtypes [32]. However, functional correlates of these anatomical subtypes of MB neurons are not known. In the current study, we investigated the spatial distribution of airflow responses in the MB in detail to see if there are functional subunits within each type of MB neuron.

To our surprise, MB neurons responded strongly to a weak airflow directed towards the antenna. The response amplitude was comparable to those evoked by odorants at high concentrations [13] while the dynamics of airflow elicited responses were more complex than those previously reported for odor evoked responses. Our results reveal for the first time strong and dynamic airflow responses in MB neurons. Detailed analysis of airflow responses revealed that each type of MB neuron responds with its own unique pattern to the airflow, suggesting functional differences between these neurons. Furthermore, we found functional subdivisions within a single type of MB neuron, raising the possibility that the MB is organized at a much finer spatial scale than previously believed.

## Results

### Different subsets of MB neurons respond with different strength and dynamics to a weak airflow stimulus

**Figure 1:**
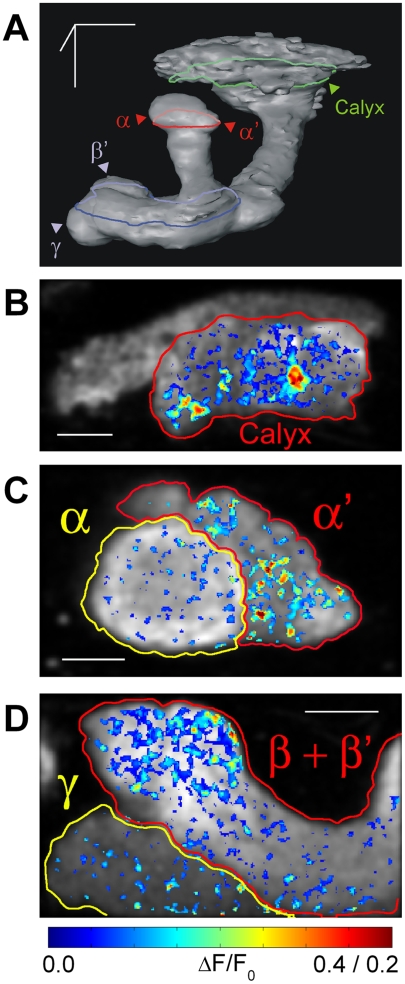
MB neurons respond strongly to weak airflow stimulus. (A) A three-dimensional reconstruction of MB neurons expressing G-CaMP driven by *OK107-Gal4*. Position of the calyx and the tips of the α, α', β', and γ lobes are shown. The β lobe is hidden behind the γ and β' lobes with this point of view. Green, red, and blue circles indicate the position of the recordings shown in (B), (C), and (D), respectively. Scale bars are 50 µm, and are positioned along the dorsal-ventral, antero-posterior, and medial-lateral axis. In the three-dimensional model, dorsal is to the top, posterior is to the back, and the lateral is to the right. (B–D) Examples of responses to a single airflow stimulation recorded from a section of the calyx (B), the vertical lobe (C), and the horizontal lobe (D). Responses are shown as a pseudo-colored ΔF/F_0_ image (range 0 to 0.4 for (B) and (D), 0 to 0.2 for (C)), and are averaged over the 6-second period after airflow on. The calyx and individual lobes are circled and identified in the figure. ΔF/F images are overlaid on a gray scale basal fluorescence image of that region. Scale bars are 20 µm for (B) and (D), and 10 µm for (C). In all figures, posterior is to the top and the lateral is to the right.

First, we used the previously characterized *OK107-Gal4* line [33] to express a genetically encoded calcium sensor, G-CaMP [34], in all three types of MB neurons. Figure 1A shows a three-dimensional reconstruction of MB neurons expressing G-CaMP driven by *OK107-Gal4* (see Materials and Methods). Using *in vivo* two-photon laser-scanning microscopy, we imaged calcium activity in response to airflow presentations from the calyx, the input region of the MB, and the vertical and horizontal lobes, the output regions of the MB. In the horizontal lobes it is sometimes difficult to separate the β lobe from the β' lobe based solely on the position of the lobe. Therefore, for experiments using *OK107-GAL4; UAS-G-CaMP* flies, responses from β lobes and β' lobes were grouped together and shown as the β+β' lobes (see below for the separation of the responses from these two lobes).

When we directed a weak airflow (3-seconds duration, 100 ml/min; see Materials and Methods) to the antenna, we found surprisingly large calcium responses in the calyx and lobes of the MB indicated by large increases in the G-CaMP fluorescence relative to the basal fluorescence level (ΔF/F_0_; see Materials and Methods). Figures 1B–D show examples of the spatial distribution of the responses in each region. The responses were distributed unevenly in each region, and stronger responses were more concentrated in the calyx, the α' lobe, and the β+β' lobes. However, since G-CaMP may not be able to detect weak activity in neurons [35], [36] and we apply a threshold to the G-CaMP fluorescence fluctuations to separate signal from noise (see Materials and Methods), lack of clear signals in some regions does not exclude the possibility that those regions are activated weakly by the airflow. Rather, the results suggest that strong responses to the airflow are distributed unevenly.

**Figure 2:**
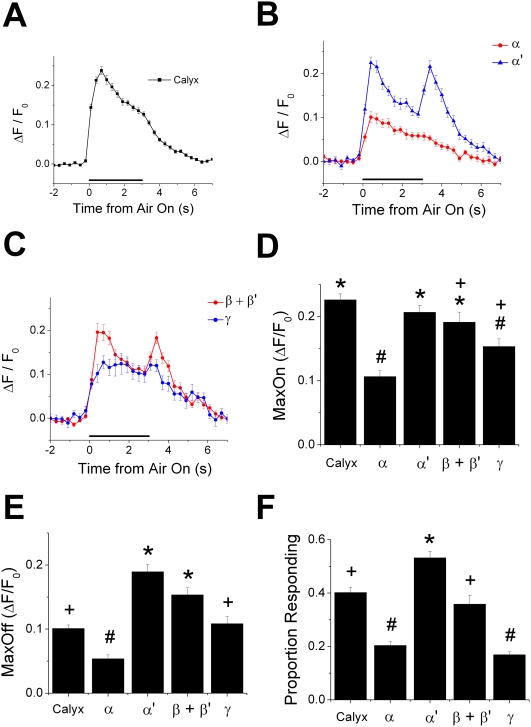
The calyx and each lobe respond with a unique pattern to airflow stimulus. (A–C) Averaged response time course (ΔF/F_0_) to a 3-second airflow stimulus recorded from calyces (A), vertical lobes (B), and horizontal lobes (C). Black horizontal bars indicate the 3-second period when airflow was directed towards the antenna. (D and E) Average amplitudes of the peak response during airflow on (D) and airflow off periods (E) (see Materials and Methods) in each region of the MB. (F) Average proportion of pixels in each region that showed significant response to airflow stimulation (see Materials and Methods). All error bars indicate the standard error of the mean (s.e.m.). Means with the same symbol (“*”, “+”, and “#”) are not significantly different from each other (P>0.05, *post hoc* Tukey HSD). Calyces, n = 33 recordings (from 8 flies), vertical lobes, n = 19 recordings (from 7 flies), and horizontal lobes, n = 16 recordings (from 7 flies).

To quantify the average time course of the airflow responses in each region of the MB, we recorded the responses from multiple depths in calyces, vertical lobes, and horizontal lobes (for the distribution of the recording depths see Fig. S1). Averaged response time courses showed that each region responds with a distinct pattern of calcium activity to the airflow turning on and off (airflow on and off) (Fig. 2A–C; for principal component analysis of the response pattern see Fig. S2). All regions showed a response to airflow on, but the response amplitudes were different among regions (Fig. 2D–E; P<1.0×10^−11^, one-way ANOVA). The calyx showed the strongest response to airflow on, and the α' lobe and the β+β' lobes responded with similar amplitudes (Fig. 2D; P>0.05, *post hoc* Tukey HSD), while the α lobe and the γ lobe response was significantly weaker than the calyx (Fig. 2D; P<1.0×10^−4^, *post hoc* Tukey HSD). On the other hand, only the α' lobe and the β+β' lobes showed a clear response to airflow off (Fig. 2A–C). These two regions had significantly larger responses to airflow off compared to the other regions (Fig. 2E; P<1.0×10^−35^, one-way ANOVA; individual comparison of the means P<0.05, *post hoc* Tukey HSD).

Except in the γ lobe that produced a unique plateau-like response without peaks, the responses to airflow on peaked and started to decay before airflow off (Fig. 2A–C). After airflow off, responses decayed further in the calyx, the α lobe, and the γ lobe, while in the α' lobe and the β+β' lobe the response peaked again before decaying (Fig. 2A–C). The time courses of all these decays were fit well with single exponential functions, and the speed of the decay was similar across regions for both the decays after airflow on (τ = 3.60, 3.91, 3.03, and 3.31 (s) for the calyx, the α lobe, the α' lobe, and the β+β' lobes), and the decays after airflow off (τ = 1.35, 1.50, 1.28, 1.21, and 2.20 (s) for the calyx, the α lobe, the α' lobe, the β+β' lobes, and the γ lobe). Faster decays after airflow off suggest that the decay of the response during the airflow on period and the off period might be caused by different mechanisms.

The proportion of pixels that showed a significant response to airflow stimulation was also different among regions (P<1.0×10^−13^, One-way ANOVA, Fig. 2F). The α' lobe had a significantly larger proportion of airflow-responding pixels compared to other regions, while the α and γ lobes had a significantly smaller proportion of airflow-responding pixels compared to other regions (P<1.0×10^−4^, *post hoc* Tukey HSD).

**Figure 3:**
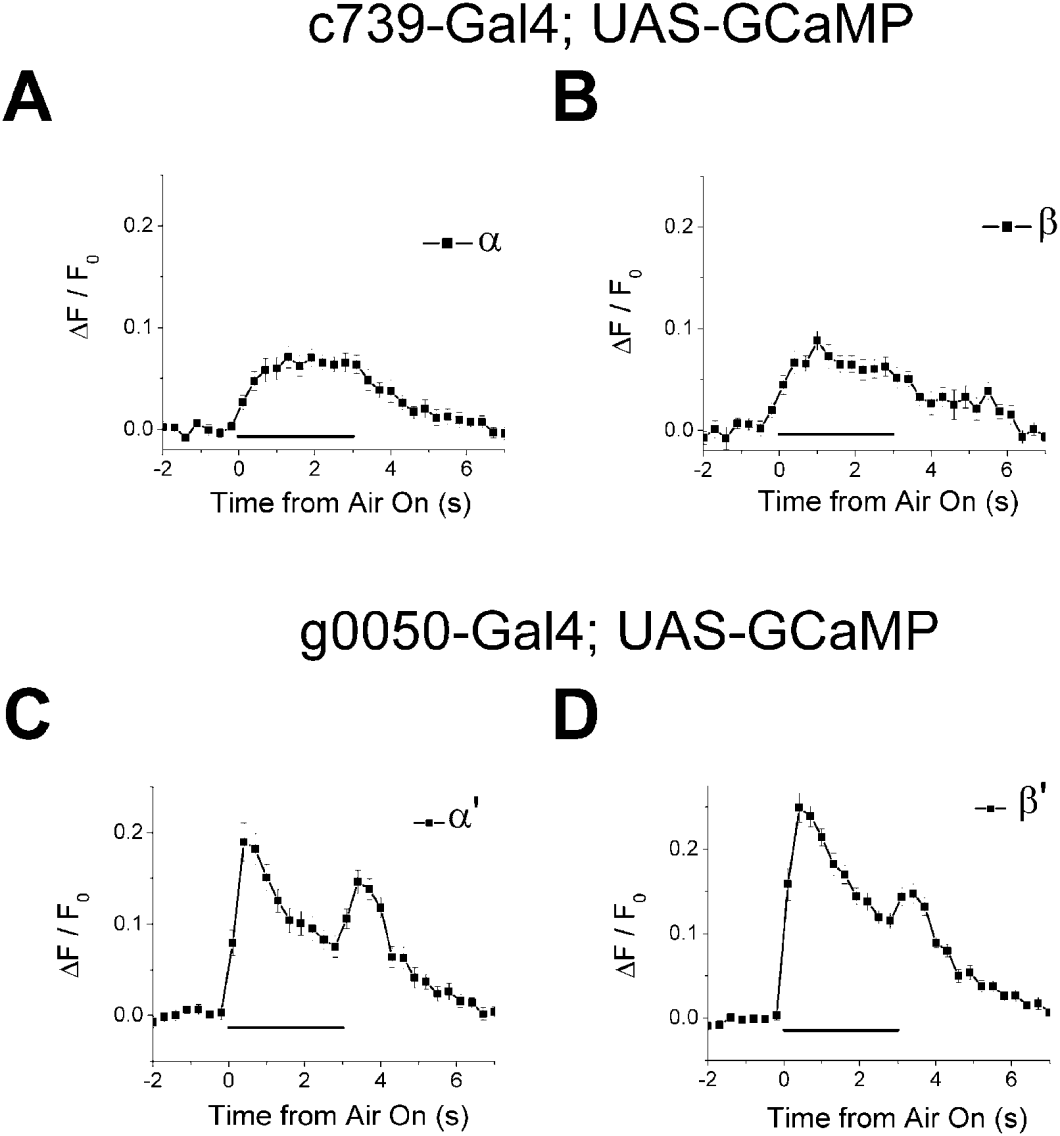
β lobes respond similarly to α lobes, while β' lobes respond similarly to α' lobes. (A–B) Averaged response time course (ΔF/F_0_) for α lobes (A) and β lobes (B) recorded using α/β neurons specific Gal4 line *c739*. α lobes and β lobes respond similarly to airflow stimulus. For α lobes, n = 18 recordings (from 7 flies), for β lobes, n = 16 recordings (from 7 flies). (C–D) Averaged response time course (ΔF/F_0_) for α' lobes (C) and β' lobes (D) recorded using α'/β' neurons specific Gal4 line *g0050*. α' lobes and β' lobes respond similarly to airflow stimulus. For α' lobes, n = 13 recordings (from 4 flies), for β' lobes, n = 24 recordings (from 6 flies). All error bars indicate s.e.m. Black horizontal bars indicate the 3-second period when airflow was directed towards the antenna.

If the airflow response properties of each lobe were determined solely by the neuronal types, the β lobe should respond similarly to the α lobe, and the β' lobe should respond similarly to the α' lobe. To test this idea, we recorded the airflow response from the β lobe and the β' lobe separately by limiting the expression of G-CaMP to either α/β or α'/β' neurons using α/β neuron specific Gal4 line *c739* [37] or α'/β' neuron specific Gal4 line *g0050* [32]. Consistent with the idea that the airflow response properties are determined by the neuronal types, the β lobe responded weakly to airflow on just like the α lobe (Fig. 3A, B; for the distribution of the recording depths see Fig. S3), and the β' lobe responded to both airflow on and off in a manner similar to the α' lobe (Fig. 3C–D). Furthermore, both the α and β lobes had a similarly smaller proportion of airflow-responding pixels compared to the α' and β' lobes (P<1.0×10^−11^, *post hoc* Tukey HSD, Fig. S4).

The airflow response recorded from α lobes and α' lobes did not change even when G-CaMP expression in these lobes was driven by different Gal4 lines (for α lobes, *c739-Gal4* (Fig. 3A) vs *OK107-Gal4* (Fig. 2B); for α' lobes *g0050-Gal4* (Fig. 3C) vs *OK107-Gal4* (Fig. 2B)). These results suggest that the airflow responses we see in these lobes are truly characteristic of each lobe and not a property of the subsets of neurons in each lobe that are driven by different Gal4 drivers, nor caused by differences in the expression level of G-CaMP in different lobes. Furthermore, small error bars for the airflow response time courses shown in Figures 2 and 3 suggest that airflow responses in each region of the MB are consistent across different recordings from different flies.

### Airflow responses in the MB can be greatly reduced by restricting the movement of the 3^rd^ antennal segment

As an initial step in identifying how airflow on and off evokes calcium responses in MB neurons, we attempted to identify the sensory organ responsible for airflow detection. In our setup, airflow was directed only towards antenna. One candidate sensory organ responsible for detecting airflow that is located in this area is Johnston's organ (JO). JO is activated by the movement of the 3^rd^ antennal segment, and has been suggested to play a role in detecting wind, acceleration, and gravity [38], [39] in addition to its well established role as a detector of near field sound [40], [41]. Since the 3^rd^ segment of the antenna moved back when the airflow was turned on and swung forward to the original position when the airflow was turned off (data not shown), we focused on the movement of the 3^rd^ antennal segment and its role in detecting airflow.

**Figure 4:**
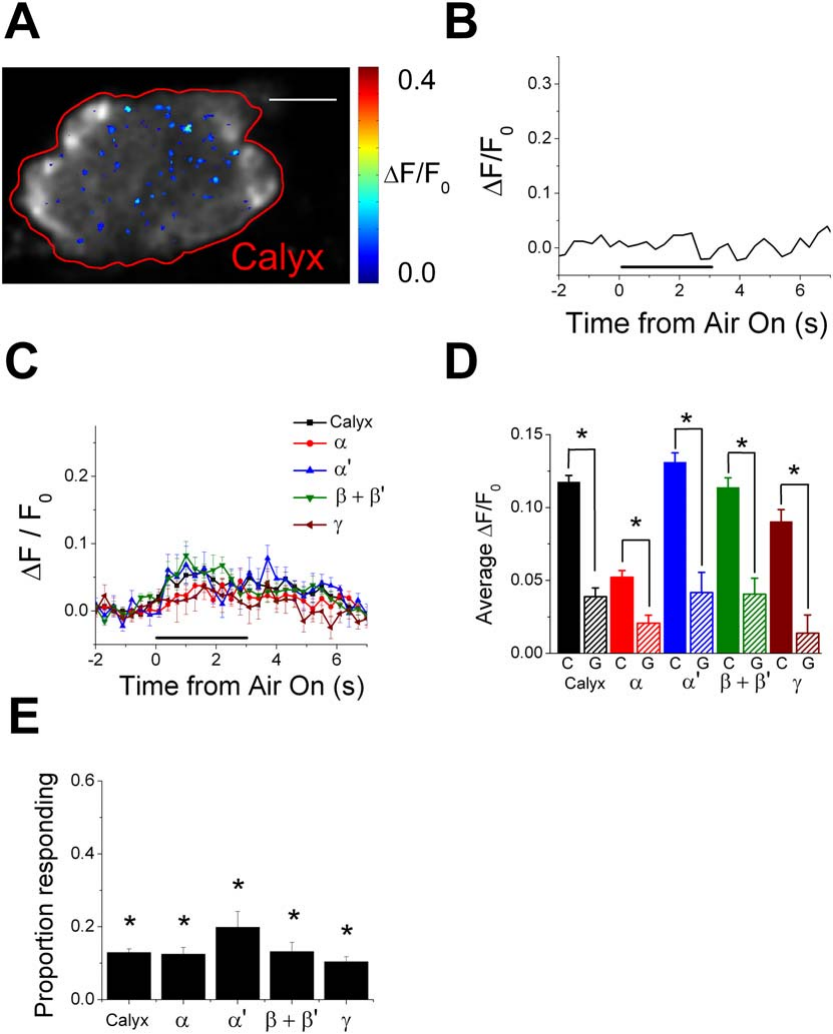
Immobilizing the 3^rd^ antennal segment significantly reduces airflow responses in the MB. (A) An example of airflow response in the calyx (circled by a red line) of a fly that had its 3^rd^ antennal segment immobilized. Responses are shown as a pseudo-colored ΔF/F_0_ image (range, 0 to 0.4) averaged over the 6-second airflow response period. Scale bar: 20 µm. Posterior is to the top and the lateral is to the right. (B) Time courses of airflow responses shown in (A). Responses are average responses of pixels that showed significant response to airflow. A black horizontal line indicates the 3-second period when airflow was directed towards the antenna. (C) Average time courses of airflow responses (ΔF/F_0_) recorded from calyces and the lobes of flies with its 3^rd^ antennal segment immobilized (mean±s.e.m.). (D) The average airflow response amplitude for each region of the MB in control (solid bars; marked “C”) and the glued (bars with diagonal lines; marked “G”) flies (±s.e.m.). Control flies are the same as those shown in Figure 2. “*” indicates means that are significantly different at P<0.01. (E) Average proportion of the airflow responding pixels in each region of the MB of flies with their 3^rd^ antennal segments immobilized (±s.e.m.). “*” indicates means that are significantly different from the control cases shown in Figure 2F. P<0.05. n for the calyces = 16 recordings (from 3 flies), vertical lobes = 9 recordings (from 3 flies), and horizontal lobes = 8 recordings (from 2 flies).

To study whether movement of the 3^rd^ antennal segment is necessary to evoke the airflow responses observed in the MB, we restricted the movement of the 3^rd^ antennal segment using non-odorant silicon adhesive (see Materials and Methods), and recorded from the MB while applying airflow to the antenna. This movement restriction greatly reduced the airflow response in the calyx (Fig. 4A, B).

We recorded airflow-evoked responses from randomly chosen locations in the calyces, vertical lobes, and horizontal lobes of flies with the 3^rd^ antennal segment immobilized, and calculated average response time courses for each region (Fig. 4C). The movement restriction greatly reduced the responses in all regions of the MB, and the difference in the dynamics of the responses we observed earlier became mostly undetectable (compare Fig. 4C with Fig. 2A–C). Furthermore, this procedure increased the variations in the amplitude and time course of the airflow responses compared to the control cases, as can be seen from the larger error bars in Fig. 4C compared to Fig. 2A–C. This may be due to differences in the extent of the immobilization of the antenna between different flies. We compared the average response amplitude (see Materials and Methods) between control flies and flies with 3^rd^ antennal segment immobilization, and found that immobilization of the 3^rd^ antennal segment significantly reduced the responses in all regions of the MB (P<0.01, unpaired two-tailed Student's *t-test*, P values corrected for multiple comparisons using Dunn-Sidak method, Fig. 4D). The immobilization of the 3^rd^ antennal segment also significantly reduced the proportion of airflow-responding pixels in all regions of the MB (P<0.05, unpaired two-tailed Student's *t-test*, P value corrected for multiple comparisons using Dunn-Sidak method, Fig. 4E compared to Fig. 2F). These results suggest that a significant portion of the airflow responses in the MB is evoked by the movement of the 3^rd^ antennal segment.

One of the concerns with antenna immobilization experiments is that the application of glue to the antenna may damage, or cover up some olfactory sensilla, and that the reduction in the airflow response may be due to a decrease in the response of olfactory sensory neurons. To exclude this possibility, we also compared odor-evoked responses between the control and antenna immobilized flies using two odorants, MCH and BA (Fig. S5). In these experiments, airflow rate to the antenna was kept constant to record odor-evoked responses in the absence of airflow-evoked responses (see Materials and Methods). Immobilization of the antenna did not change the amplitude of the response to BA and MCH in all regions of the MB (P>0.05, unpaired two-tailed Student's *t-test*, P value corrected for multiple comparisons using Dunn-Sidak method, Fig. S5). The proportion of pixels responding to BA and MCH also did not differ between control flies and antenna immobilized flies in all regions of the MB (P>0.05, Fig. S5). This further suggests that airflow-evoked responses are caused by mechanical movements of the 3^rd^ antennal segment. Small residual airflow responses from the MB of the flies with glued antenna may be a result of insufficient immobilization of the antenna due to the elastic nature of the silicon adhesive, or may be an indication that there are other sources that evoke the airflow responses in the MB.

### Responses in the calyx are homogenous, but the lobes have functional subdivisions that respond differently to airflow on and off

**Figure 5:**
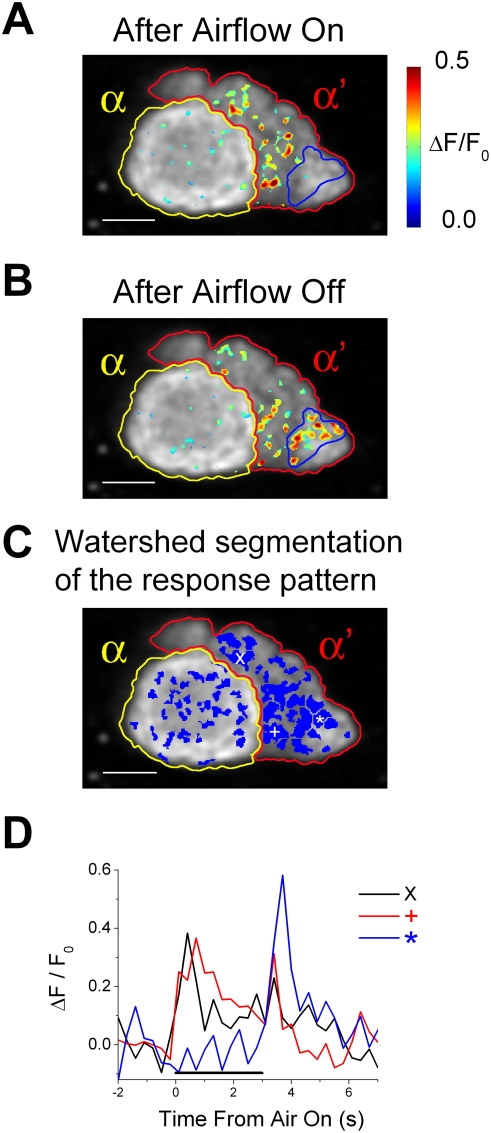
Different areas inside the α' lobe respond differently to airflow on and off. (A–B) Examples of the responses after airflow on (A) and off (B) recorded from a section of the vertical lobe. This section is the same as the one shown in Figure 1C. Responses are shown as a pseudo-colored ΔF/F_0_ image (range 0 to 0.5) averaged over the 1-second period after airflow on (A) or off (B). In each figure, ΔF/F_0_ image is overlaid over a gray scale basal fluorescence image of that region. Regions of interests are each circled and labeled in the figure (yellow circles = α lobe and red circles = α' lobe). Blue circles indicate a region in the α' lobe that did not respond to airflow on but responded strongly to airflow off. Scale bars are 10 µm. In all figures, posterior is to the top and the lateral is to the right. (C) Watershed segmentation of the spatial pattern of airflow response for the same section of the vertical lobe shown above. Watershed segmentation divides the airflow responding areas into smaller “patches” that share a common local peak of response. Symbols, “x”, “+”, and “*” indicate the positions of the patches that have response time courses shown in (D). Scale bars and circles surrounding the regions of interests are the same as in (A). (D) Time courses of the airflow responses recorded from the patches marked in (C). Responses are shown as a ratio of fluorescence change over the basal fluorescence (ΔF/F_0_), and are average responses of pixels in the patches that are marked. Black horizontal bars indicate the 3-second periods when the airflow was directed towards the antenna.

Next, we investigated how different spatial locations inside the calyces and lobes responded to airflow on and off. We were particularly interested in studying whether the areas responding to airflow on were the same as the areas responding to airflow off. Figures 5A and 5B show examples of areas in a section of the vertical lobe that were responsive immediately after airflow on (Fig. 5A) and off (Fig. 5B). In the α' lobe, strong responses were seen after both airflow on and off. However, the areas responding to airflow on were different from those that responded to airflow off (Fig. 5A, B; the area circled by the blue line responded only to airflow off). This result suggests that α' lobes may be divided into functional subdivisions that respond differently to airflow on and off.

To study how individual subdivisions within the calyx and the lobes respond to airflow on and off in more detail, we took each recording and divided the airflow-responding areas into smaller “patches” that corresponded to the local hot spots of the response. This was achieved by applying watershed segmentation to the spatial pattern of the airflow response calculated for each recording (see Materials and Methods). Figure 5C shows the patches made by applying this method to the airflow-responding areas in the sections of the vertical lobe illustrated in Figures 5A and 5B. The response time courses for 3 individual patches from the α' lobe are plotted in Figure 5D. Some patches responded to both airflow on and off (patches marked with “x” and “+”), while others responded only to airflow off (patch marked with “*”), confirming that different subdivisions within the α' lobe respond differently to airflow on and off (Fig. 5D). Based on their sizes, these patches are likely to be composed of multiple axons and axon terminals (for the distribution of the patch area see Fig. S6).

**Figure 6:**
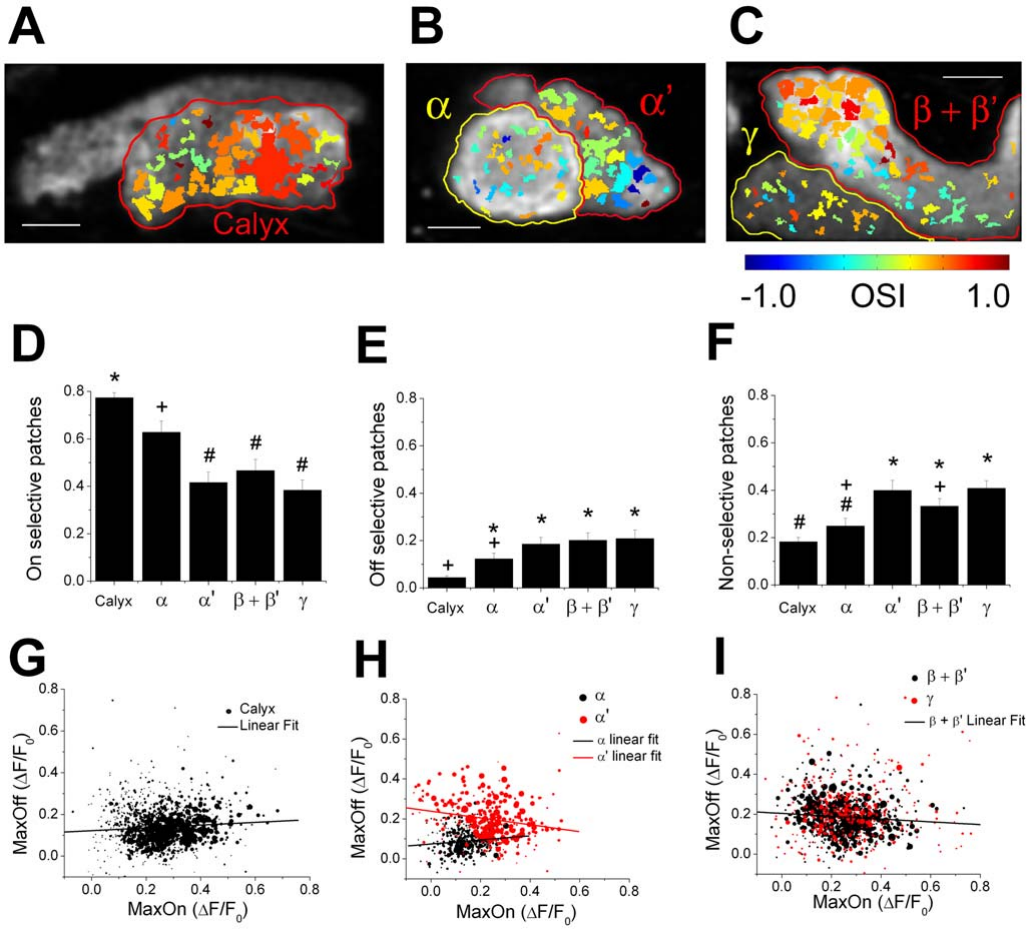
Patches from different regions of the MB show different responses to airflow on and off. (A–C) Patches obtained by watershed segmentation of the average response image shown in Figures 1B–1D color coded with OSI (see Materials and Methods). Scale bars, circles surrounding the regions of interests, and the orientation of the figures are the same as in Figures 1B–1D. (D–F) The average proportion of the airflow-responding areas occupied by patches highly selective to airflow on (OSI>0.2; (D)), highly selective to airflow off (OSI<−0.2; (E)), and those that are non-selective (–0.2 = <OSI = <0.2; (F)) are shown for each region of the MB (±s.e.m.). Means with the same symbol (“*”, “+”, and “#”) are not significantly different from each other (P>0.05, *post hoc* Tukey HSD). (G–I) All the patches recorded from calyces (G), vertical lobes (H), and horizontal lobes (I) are plotted against their maximum response after airflow on (MaxOn) and off (MaxOff) (see Materials and Methods). The area of each circle in the figure is proportional to the actual area of each patch (circles in the legends are equivalent to 100 µm^2^ for calyces, 50 µm^2^ for vertical lobes, and 75 µm^2^ for horizontal lobes). n for Calyces = 1510 patches (from 33 recordings in 8 flies), α lobes = 375 patches (from 19 recordings in 7 flies), α' lobes = 297 patches (from 19 recordings in 7 flies), β+β' lobes = 643 patches (from 16 recordings in 7 flies), and γ lobes = 332 patches (from 16 recordings in 7 flies). Solid black lines in (G), (H), and (I) indicate linear regression lines for patches in the calyx, the α lobe, and the β+β' lobe, respectively. The solid red line in (H) indicates the linear regression line for patches in the α' lobe.

To quantify how selectively the individual patches respond to airflow on and off, we calculated an On-Off Selectivity Index (OSI) for each patch (see Materials and Methods). The OSI represents the difference between responses to airflow on and off relative to the total response to the airflow, and ranges from 1 (responds only after airflow on) to –1 (responds only after airflow off). To visualize the distribution of the OSI in each region of the MB, we color-coded individual patches with this index for a section of the calyx (Fig. 6A), the vertical lobe (Fig. 6B), and the horizontal lobe (Fig. 6C). The calyx had patches with mostly warm colors indicating a high and homogeneous OSI (Fig. 6A). However, the lobes had patches with very diverse colors indicating an OSI that varied greatly from one subdivision to the other (Fig. 6B–C).

For statistical comparison, we quantified the proportion of the airflow-responsive areas that were highly selective to airflow on (OSI>0.2; Fig. 6D), highly selective to airflow off (OSI<−0.2; Fig. 6E), and relatively non-selective (OSI between –0.2 and 0.2; Fig. 6F) (for the statistical comparison of the distribution of the OSI for patches in the calyces and lobes see Fig. S7). As expected from their average response properties (Fig. 2), the calyx and the α lobe had a larger proportion of airflow-responsive areas covered by patches that were highly selective to airflow on compared to the other lobes (P<0.05 One-way ANOVA, *post hoc* Tukey HSD; Fig. 6D). However, even the α' lobe and the β+β' lobes, that showed strong responses to airflow off on average, had a large proportion of airflow-responding areas (around 40%) that were highly on-selective, further suggesting that the response selectivity in these lobes is highly heterogeneous. As for the regions highly selective to airflow off, the calyx had a smaller proportion compared to all lobes except the α lobe (P<0.05 One-way ANOVA, *post hoc* Tukey HSD; Fig. 6E). For the regions showing non-selective responses, the situation was similar to those regions highly selective to airflow off, except that the α lobe also had significantly less proportion of area showing non-selective responses compared to the α' and γ lobes (P<0.05, One-way ANOVA, *post hoc* Tukey HSD; Fig. 6F). Overall, these analyses confirm and statistically verify the idea that the calyx responds homogenously to airflow on while different areas within each lobe respond differently to airflow on and off.

The OSI quantifies the relative relationship between the responses after airflow on and off. We also compared the absolute amplitudes of the responses after airflow on and off between the patches recorded from calyces, vertical lobes, and horizontal lobes. This was done by plotting each patch in the region according to its maximum response after airflow on and off (Fig. 6G–I). The patches from the calyx clustered tightly in a narrow range where the responses after airflow on are large and the responses after airflow off are small (Fig. 6G). Patches from the α lobe also clustered in a relatively narrow region but showed smaller responses after airflow on compared to the calyx (Fig. 6H). On the other hand, patches from the α', β+β', and γ lobes spread out widely, reflecting the large variability in the airflow response properties of the patches in these lobes (Fig. 6H–I). In these lobes, the patches did not form discrete clusters, suggesting that there is no clear divide between the patches that respond strongly after airflow on and those that respond strongly after airflow off.

In the calyx and the α lobe, the responses after airflow on and off were weakly correlated (for the calyx, Pearson's correlation (r) = 0.0788, P<0.01; the α lobe, r = 0.1132, P<0.05; Fig. 6G–H). This weak positive correlation is probably due to the airflow on responses that were decaying but still present during the period after airflow off. Interestingly, in the α' lobe and the β+β' lobes, the responses after airflow on and off were negatively correlated (For the α' lobe, r = −0.205, P<0.001; The β+β' lobes, r = −0.083, P<0.05; Fig. 6H–I). Strong negative correlation between the responses after airflow on and off in the α' lobe was observed consistently across different flies (statistically significant (P<0.01) negative correlation was seen in 4 out of 7 flies when tested individually. The rest of the flies also showed a tendency for a negative correlation). Patches in the γ lobe did not show statistically significant correlation between the response after airflow on and off.

### Patches that show high selectivity to airflow off are spatially clustered in a stereotypic location in the α' lobe

**Figure 7:**
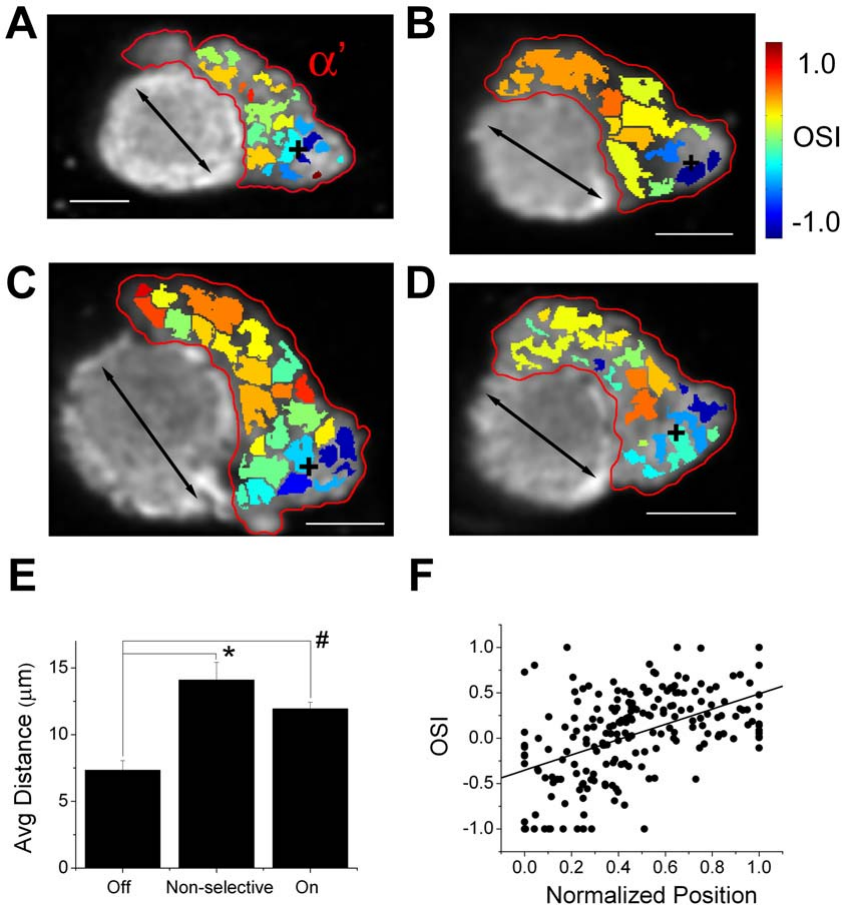
Airflow off selective patches are spatially clustered and are located in a stereotypic location. (A–D) Examples of the distribution of the OSI in recordings made near the tip of the α' lobe of 4 different flies. Patches were generated by watershed segmentation, color-coded by OSI, and overlaid on a gray scale basal fluorescence image recorded from that region. Black crosses indicate the center of gravity for the patches that are highly selective to airflow off (OSI<–0.2). Red circles indicate α' lobes. Black arrows indicate the anterolateral to posteromedial axis of the α' lobe estimated for each recording. In all figures, posterior is to the top and the lateral is to the right. Scale bars are 10 µm. (E) Average distances among the patches that are highly selective to airflow on, off, and those that are non-selective (±s.e.m., n = 11 recordings (from 6 flies)). “*” and “#” indicate means that are significantly different at P<1.0×10^−4^ and P<1.0×10^−3^, respectively. (F) OSI of each patch recorded near tips of α' lobes plotted against its normalized position along the anterolateral to posteromedial axis for each section of the α' lobe (n = 205 patches (from 11 recordings in 6 flies)). The solid black line indicates the linear regression line.

Because of the relatively strong negative correlation between the responses after airflow on and off in the patches from the α' lobe, we looked at the spatial distribution of the OSI in the α' lobe in more detail. We noticed that near the tip of the α' lobe (5 to 15 µm from the tip) there were areas in the anterolateral part of the α' lobe that responded selectively to airflow off (Fig. 7A–D; black crosses indicate the center of gravity for the patches with OSI<−0.2 in each recording). This cluster of patches that responded selectively to airflow off was found consistently in all the recordings made near the tip of α' lobes. The average distances among patches highly selective to airflow off (OSI<−0.2) calculated from all the recordings made near the tip of the α' lobes were significantly shorter than the average distances among the patches highly selective to airflow on (OSI>0.2) (P<0.001, *post-hoc* Tukey HSD, Fig. 7E), or the average distance among the patches that were non-selective (OSI from –0.2 to 0.2) (P<1.0×10^−4^, *post-hoc* Tukey HSD, Fig. 7E). This confirms that the patches highly selective to airflow off are spatially clustered compared to the other patches. Patches highly selective to airflow on were spatially closer to each other compared to those that were non-selective, but this difference was not statistically significant (P>0.182, post-hoc Tukey HSD, Fig. 7E).

Figures 7A–D show not only a clustering of highly off-selective patches but also a consistent trend for the OSI of the patches to increase as the position of the patch moves along the anterolateral to posteromedial axis of α' lobes. To statistically verify this trend, we estimated the anterolateral to posteromedial axis of the α' lobe in each recording made near the tip of the α' lobe (see Materials and Methods), and plotted the OSI of each patch against its position along this axis (Fig. 7F; estimated axes are shown by black arrows in Fig. 7A–D). Position along this anterolateral to posteromedial axis was normalized so that the position of the most anterolateral and most posteromedial patch in each recording became 0 and 1 respectively. There was a positive correlation between the OSI of each patch and its normalized position (r = 0.491, P = 7.0×10^−14^, Fig. 7F), suggesting that indeed patches become more selective to airflow on as the position moves more posteromedial (for the correlation between the normalized position of patches and the maximum airflow on and off responses see Fig. S8). This positive correlation was seen consistently across the flies (when tested individually, 5 out of 6 flies showed statistically significant correlation (P<0.05). One fly showed a trend of positive correlation that was not statistically significant.). However, this linear trend seemed to break down at the more anterolateral portion of α' lobes where highly off-selective patches exist, further suggesting that the dorsal lateral portion of the α' lobe may be functionally distinct from the other regions.

### Recordings from different subsets of α'/β' neurons confirm the stereotypic localization of the strong airflow off responses in the α' lobe

**Figure 8:**
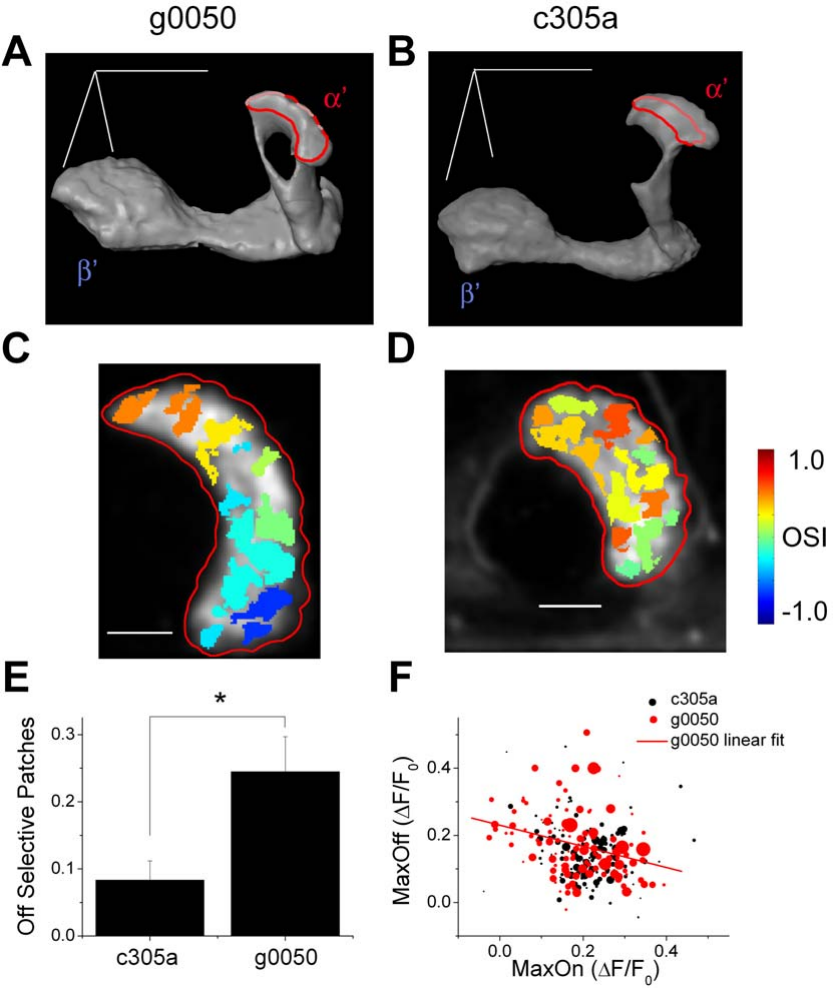
Airflow off regions in the α' lobe can be separated using two GAL4 lines. (A–B) A three-dimensional reconstruction of the vertical and horizontal lobes of MB neurons expressing G-CaMP driven by two α'/β' neurons specific GAL4 lines *g0050* (A) and *c305a* (B). Red circles indicate positions of the recordings shown in (C) and (D). Scale bars are 50 µm and are positioned along the dorsal-ventral, antero-posterior, and medial-lateral axis. In the three-dimensional model, dorsal is to the top, posterior is to the back, and the lateral is to the right. (C–D) Examples of the distribution of the OSI in sections of α' lobes from a *g0050-Gal4; UAS-G-CaMP* (C) and a *c305a-Gal4; UAS-G-CaMP* (D) flies. Both sections were located between 5 to 15 µm from the tip of the α' lobe. Patches were generated by watershed segmentation, color-coded by OSI, and overlaid on gray scale basal fluorescence images of that region. Red circles indicate α' lobes. Scale bars are 10 µm. Posterior is to the top, and the lateral is to the right. (E) Average proportion of the airflow-responsive area occupied by patches highly selective to airflow off (OSI<–0.2) in the α' lobe of *c305a-Gal4; UAS-G-CaMP* and *g0050-Gal4; UAS-G-CaMP* flies (mean±s.e.m.). n = 8 recordings each (from 4 flies for *c305a-Gal4; UAS-G-CaMP* and from 3 flies for *g0050-Gal4; UAS-G-CaMP*). Only recordings made within 5 to 15 µm from tips of α' lobes were included. “*” indicates a statistically significant difference at P<0.05. (F) Patches recorded from α' lobes of *c305a-Gal4; UAS-G-CaMP* and *g0050-Gal4; UAS-G-CaMP* flies plotted against its maximum airflow on and off responses (n for *c305a-Gal4; UAS-G-CaMP* flies = 127 patches (from 8 recordings in 4 flies), *g0050-Gal4; UAS-G-CaMP* flies = 112 patches (from 8 recordings in 3 flies)). The area of the circle is proportional to the area of each patch. The circle in the legend represents 50 µm^2^. Only recordings made within 5 to 15 µm from the tip of the α' lobe were included. The red solid line indicates the linear regression line.

To further confirm the stereotypic spatial clustering of the strong airflow off responses in the α' lobes, we used two previously characterized GAL4 lines that drive expression of transgenes in different subgroups of α'/β' neurons. *g0050* drives expression of transgenes in most of the α'/β' neurons, while *c305a* drives expression of transgenes only in the posterior half of the α' lobe [30], [32] (Fig. 8A–B). If the patches responding selectively to airflow off are really clustered in the anterolateral part of the α' lobe, they should appear in a similar part of the α' lobe in *g0050-Gal4; UAS-G-CaMP* flies but should be absent in *c305a-Gal4; UAS-G-CaMP* flies. We expressed G-CaMP using these two α'/β' specific GAL4 lines and measured airflow evoked responses from the α' lobes using the same procedures as before. Consistent with our hypothesis, we found that in *g0050-Gal4; UAS-G-CaMP* flies, patches highly selective to airflow off were spatially clustered in the anterolateral part of the α' lobe (Fig. 8C), while *c305a-Gal4; UAS-G-CaMP* flies lacked clusters of patches that were highly selective to airflow off (Fig. 8D). Statistical comparison of the proportion of α' lobes that were highly selective to airflow off (OSI<−0.2) confirmed that *c305a-Gal4; UAS-G-CaMP* flies had a significantly smaller proportion of patches selective to airflow off compared to *g0050-Gal4; UAS-G-CaMP* flies (P<0.05, unpaired two-tailed Student's *t-test*, Fig. 8E; for the statistical comparison of the distribution of the OSI of the patches see Fig. S7). These results also eliminate the possibility that the spatial clustering of patches highly selective to airflow off observed in *OK107-Gal4; UAS-G-CaMP* flies were due to a selective labeling of the airflow off responding neurons in these areas with *OK107-GAL4*.

To compare the absolute amplitudes of the response to airflow on and off between the patches recorded from the α' lobes of *g0050-Gal4; UAS-G-CaMP* and *c305a-Gal4; UAS-G-CaMP* flies, we plotted each patch according to its maximum response after airflow on and off (Fig. 8F; for the distribution of the patch area see Fig. S9). As expected, *c305a-Gal4; UAS-G-CaMP* flies had fewer patches that responded strongly to airflow off compared to *g0050-Gal4; UAS-G-CaMP* flies. In *g0050-Gal4; UAS-G-CaMP* flies, the patches showed a negative correlation between airflow on and off responses as in *OK107-Gal4; UAS-G-CaMP* flies (r = −0.297, P<0.01, Fig. 8F). This negative correlation was observed consistently across the flies (When tested individually, 2 out of 3 flies showed a statistically significant negative correlation (P<0.05), while the other fly showed a trend of negative correlation.). On the other hand, negative correlation was not observed in *c305a-Gal4; UAS-G-CaMP* flies (P>0.1), presumably due to the lack of patches highly selective to airflow off.

## Discussion

### Strong response to a weak airflow

This study provides the first evidence that the *Drosophila* MB, a higher olfactory center, responds dynamically to airflow stimuli. We found that even a very weak airflow stimulus can evoke surprisingly strong responses in different regions of the MB. In the calyx, the amplitude of the responses to airflow was comparable to those evoked by odorants at high concentrations as shown in a previous study [13]. Since G-CaMP cannot detect weak activation of neurons [35], [36] and we apply a pixel-by-pixel threshold to our G-CaMP signals (see Materials and Methods), the results shown in this study are a conservative estimate of activity levels, reflecting strong activition in response to airflow. It is possible that even regions that did not have airflow responding pixels as defined in this study may still be responding weakly to airflow, and the airflow evoked activities in the MB may be more widespread than shown here. The airflow we directed towards the fly antenna is equivalent to a wind speed of 1.2 m/sec, which corresponds to the lightest wind in a Beaufort scale used by meteorologists, and is close to the maximum air speed for free-flying flies that encountered an odor plume [42]. This means that the MB is continuously receiving a very strong airflow input while the fly is in a natural environment, and suggests that activation related to airflow must play an important role in the normal function of the MB.

### Transformation from homogenous input in the calyx to characteristic outputs in the lobes

The calyx, the input region of the MB, responded only to airflow on (Fig. 2A) and its response properties were quite homogenous even when the calyx was subdivided into smaller patches (Fig. 6). However, when we measured the airflow responses in the output regions of the MB, we found that each lobe responds differently to airflow on and off (Fig. 2 and 3). On average, the α' and β' lobes responded to both the airflow on and off, while the α, β, and γ lobes responded only to the airflow on (Fig. 2, 3, and 8). The α' and β' lobes also had significantly more proportion of pixels responding to the airflow compared to the α, β, and γ lobes (Fig. 2F and S4), and the amplitudes of the response were also larger in these lobes (Fig. 2 and 3).

This change from homogenous responses at the input region to neuronal type-specific responses at the output region is not simply due to a mixing of signals from neurites of different types of MB neurons in the calyx since recordings from calyces of flies that have G-CaMP expressed specifically in the α/β neurons or the α'/β' neurons also show airflow responses similar to the calyces of flies that have G-CaMP expressed in all types of MB neurons (data not shown). Rather, it suggests that airflow information is processed differently in each type of MB neuron. However, due to the limitation in the sensitivity of G-CaMP and the spatial resolution of our recordings, we cannot completely rule out the possibility that, below our signal detection threshold, each type of MB neuron is responding differently even in the calyx.

Possible functional differences between different types of MB neurons have been suggested many times [14], [26], [27], [28], [29], [30], [31]. It is interesting to note that the α'/β' neurons, a type of MB neuron that is essential for the acquisition and stabilization of olfactory memory [30] and shows an early trace of olfactory memory [31], were the ones that responded most strongly to airflow on and off. These neurons also respond strongly to olfactory stimuli [31], [43]. Perhaps the property of these neurons to respond strongly to stimuli of different modalities enables them to integrate olfactory information with other sensory information and aids in the formation of associative memory.

### Functional subdivisions and their stereotypic distribution within a single type of MB neuron

When we divided responses in each lobe into smaller “patches” by watershed segmentation (Fig. 5 and 6), we found that the α' lobe has a smaller subdivision that responds specifically to airflow off (Fig. 7 and 8). This subdivision within the α' lobe was located in the anterolateral part of the lobe, and this location was conserved across flies, even when a different Gal4 driver was used (Fig. 8). Furthermore, we found that in the α' lobe, patches become more selective to airflow on as the position moved along the anterolateral to posteromedial axis of the α' lobe (Fig. 7F). The fact that the subdivision organization was conserved even when G-CaMP was expressed at different levels in different subsets of α'/β' neurons by several Gal4 lines suggests that these subdivisions are not artificial divisions that are made by differences in G-CaMP expression levels or other artifacts. Rather, these results raise the possibility that the MB is functionally organized at a much finer spatial scale than anatomically defined lobes. To the best of our knowledge, this is the first evidence to suggest the existence of stereotypic functional subdivisions within a single lobe. The patches in α' lobes of *OK107-Gal4; UAS-G-CaMP* flies had a median area of 9 µm^2^ (Fig. S6). Since most axon terminals of MB neurons seem to be ∼1 µm in diameter [44], each patch must consist of multiple axon terminals and axons. Further studies are necessary to understand how these groups of axon terminals and axons are arranged to form the fine stereotypic organizations we observed.

Different patches in the β +β' and γ lobe also responded very differently to the airflow stimulus (Fig. 6). Although we concentrated on the fine organization of the α' lobe in this study, future detailed studies in other lobes may reveal functional subdivisions in these lobes as well. Anatomical studies have shown that α/β neurons can be divided further into three subtypes according to their birth order, and that each subtype projects its dendrites into different regions of the calyx [32], [45]. Further studies are necessary to see if these anatomical subdivisions show functionally distinct responses as well.

Although calcium imaging has the ability to localize calcium activity with good spatial resolution (with a caveat of thresholding the activity at certain level), it lacks the temporal resolution necessary to capture the detailed activity pattern of the neurons. In our study, the temporal resolution was limited to 3.3 Hz, and the airflow onset and offset was not coincident with the start of the frame acquisition making it difficult to measure the precise dynamics of the response. Future electrophysiological studies with much finer temporal resolution may uncover even more functional subdivisions within the MB.

### Neural pathways to bring airflow information to the MB

Results from the antenna immobilization experiments suggest that a significant part of the airflow responses observed in the MB is caused by the mechanical movement of the 3^rd^ antennal segment (Fig. 4). This suggests that the sensory neurons of JO may be involved in the detection of the airflow. Although JO is well known for its role in detecting near-field sound [40], a recent study has shown that some of the neurons in JO are located in positions that allow them to be maximally activated with a front-back movement of the 3^rd^ antennal segment rather than the rotational movement induced by near-field sound [39]. Our airflow stimulation causes front-back movement of the 3^rd^ antennal segment, consistent with the idea that these neurons may be mediating the responses to the airflow.

However, at present, there are no anatomical data to link sensory neurons in JO to the MB. JO neurons project mainly to the antennal mechanosensory and motor center and to a lesser extent the ventrolateral protocerebrum and the subesophageal ganglion [39]. A neural pathway that connects these areas to the MB is currently unknown. On the other hand, calcium imaging from PNs show that they also respond strongly to the airflow stimulus used in the present study, suggesting that at least part of the airflow information to the MB is conveyed by PNs (A.M. unpublished observation). This idea is consistent with a previous report that 1/3 of PNs were responsive to changes in airflow even when no olfactory stimulus was used [46]. Activation of PNs by the airflow stimulus seems to indicate that olfactory sensory neurons may be involved in this response, but results from antenna immobilization studies (Fig. 4 and Fig. S5) are inconsistent with the idea that the airflow-evoked responses we observed were a simple olfactory response. Furthermore, the dynamics of airflow responses in the MB differs greatly from previously reported olfactory responses in the MB [12], [13], [14]. Direct recordings from different candidate sensory neurons during airflow stimulation and more anatomical studies are necessary before reaching a full understanding of the complete pathway that carries airflow information to the MB.

### Possible functional roles of airflow responses in the MB

What is the functional role of the surprisingly strong and dynamic airflow responses we found in the MB? One possible role may be odor source localization. The disparity in responsiveness to airflow on and off exhibited by different sets of MB neurons (Fig. 7 and 8) may allow a stationary fly to distinguish a head wind (antenna moving in an airflow on direction) from a tail wind (antenna moving in an airflow off direction), and provide directional cues for odors that are carried by the wind. It is also possible that when an animal is flying in a natural environment, the MB on different sides of the brain may receive different amounts of airflow input depending on the relative wind direction, and this may allow the fly to localize the odor source. Another possibility is that the responses to airflow on and off provide MB neurons with a burst of excitation that enables them to respond to weak olfactory inputs in a reliable and synchronous manner. Future studies on how olfactory and airflow information interact in different functional subdivisions of the MB should give us a better understanding of how the *Drosophila* MB, and higher associational brain areas in general, integrates and associates different sensory stimuli.

## Materials and Methods

### Transgenic flies

Flies carrying transgene for a genetically encoded calcium sensor *UAS-G-CaMP1.3* [13] or *UAS-G-CaMP1.6* [36] were crossed with GAL4 lines that have been previously shown to drive the expression of transgenes in the MB. *OK107-Gal4* drives expression in all three subtypes of MB neurons [33], while *c739-Gal4* drives expression only in α/β neurons [37]. *g0050-Gal4* [32] and *c305a-Gal4* [30] drive expression of transgenes specifically in α'/β' prime neurons, but *g0050-Gal4* drives expression in a larger fraction of α'/β' neurons [32]. The offspring from the crosses (*G-CaMP×MB Gal4 lines*) were used for the *in vivo* fly imaging. *OK107-Gal4* was crossed with *UAS-G-CaMP1.3*. All other lines were crossed with *UAS-G-CaMP1.6*. Compared to G-CaMP1.3, G-CaMP1.6 has larger fluorescence at rest, and shows a larger and faster fluorescence change in response to neural activity at the Drosophila larval neuromuscular junction [36]. Because of these differences, comparisons of the G-CaMP signals were done only within the same type of G-CaMP.

### *In vivo* two-photon imaging

Flies were prepared for *in vivo* imaging as described previously [13] with some modifications. Briefly, a fly was immobilized in a plastic micropipette tip with its head exposed. A plastic coverslip with a small window was placed over the head and was sealed onto the fly around the edges of the window with a silicon elastomer adhesive (Kwik-Sil, World Precision Instruments, Sarasota, FL). After covering the head with adult fly saline [103 mM NaCl, 3 mM KCl, 1.5 mM CaCl_2_, 4 mM MgCl_2_, 26 mM NaHCO_3_, 1 mM NaH_2_PO_4_, 8 mM trehalose, 10 mM glucose, and 5 mM N-Tris (hydroxymethyl) methyl-2-aminoethanesulfonic acid, pH 7.1, 356 mOsm] [47], a small hole was cut through the cuticle to make the MB accessible for imaging. After removing the cuticle, fly brains were covered with 2% agarose (Sigma, St Louis, MO) to reduce the movement of the brain. During experiments, fly brains were superfused at a rate of 2 ml/min with the above-mentioned fly saline that was gassed with 95% O_2_ and 5% CO_2_.

For all imaging, a custom-built two-photon laser scanning microscope was used as described previously [13]. The objective used was a 60x water-immersion lens (0.9 NA, Olympus America, Melville, NY). Scanning was controlled by Fluoview software (Olympus America, Melville, NY). The light source was a Chameleon Ti:Sapphire laser (Coherent, Santa Clara, CA) tuned to wavelength (λ) 910 nm. Images were acquired at a rate of 0.3 seconds per frame.

### Airflow stimulation

Airflow stimulation was applied to the antenna of a fly through a glass micropipette (ID: 1.23 mm) located 3 mm from the antenna. The position and the angle of the head of the fly were adjusted prior to fixing the fly with silicon adhesive so that the 3^rd^ segment of the antenna was placed perpendicular to the airflow and the dorsal side of the head was facing upwards. An airflow rate of 100 ml/min was used throughout the experiment. This corresponds to a wind speed of 1.2 m/sec at the end of the glass micropipette that delivers the airflow. Compressed pure medical air (General Welding Supply, Westbury, NY) was used as an air source to avoid contamination of the air with odorants. The speed of airflow was controlled by a GFC mass flow controller (Aalborg, Orangeburg, NY). A solenoid valve (NResearch, West Caldwell, NJ) was placed behind the flow controller to direct the airflow to or away from the fly. Before the stimulation, airflow was directed away from the fly into a vacuum. There was a 3 min interval between successive airflow stimulations to avoid possible habituation of the responses. At this interval, we found no habituation of the responses. The speed of airflow and the timing of the air delivery were controlled by a custom program written in MatLab (MathWorks, Natick, MA).

### Odor Stimulation

To deliver odorants to the fly's antenna with minimal change in the airflow speed, we supplied continuous airflow (450 ml/min) to the fly's antenna throughout the experiment. During odor delivery, a solenoid valve redirected a portion of airflow (150 ml/min) to a vial that contained an odorant diluted in mineral oil. This redirected airflow was merged with main airflow and presented to the fly's antenna. 4-methylcyclohexanol (MCH) and Benzaldehyde (BA) was diluted 1∶1000 and 1.5∶1000 fold in mineral oil respectively, giving a final concentration of 1.0×10^−3^ for MCH and 5.0×10^−4^ for BA. Odorants were presented for 3 seconds. There was a 3-min interval between the presentations.

### Immobilization of the 3^rd^ antennal segment

To restrict the movement of the 3^rd^ antennal segment against the 2^nd^ antennal segment, a non-toxic and non-odorant silicon elastomer adhesive (Kwik-Sil, World Precision Instruments, Sarasota, FL) was used to glue down the arista to the side of the fly head. The same silicon adhesive was also used to glue the joint between the 3^rd^ and the 2^nd^ antennal segment. Care was taken not to cover the olfactory sensilla located in the 3^rd^ antennal segment.

### Image Analysis

All image analyses were performed with a custom program written in Matlab (Mathworks, Natick, MA). The ΔF/F_0_ images were generated as described previously with some modifications [13]. Briefly, images acquired were first smoothed with Gaussian filter (7×7, σ = 2), corrected for photobleaching, and the average dark noise was subtracted. Small movements of the brain in the x-y direction during the image acquisition were corrected using a custom program. This program moves the images in the x-y direction and finds coordinates that minimize the mean-square difference between the pixel intensities of particular frame and the reference image (average of all frames acquired before airflow stimulation). After the filtering and corrections, frames acquired before the onset of airflow stimulation (16 frames) were averaged and this average baseline image was subtracted from all frames to form ΔF images. Then, for each pixel in the region of interest, the standard deviation (SD) of the baseline fluorescence fluctuation was calculated for the period before the airflow stimulation. To separate airflow evoked signals from noises, only pixels that showed fluorescence change larger than 3×SD of its baseline fluctuations during the 6-second period after the airflow on were considered as airflow responding pixels. All other pixels were removed from ΔF images. This pixel-by-pixel thresholding procedure was necessary since different pixels have different baseline fluctuations due to brain movements. This procedure has been used in our previous studies to separate odor evoked signals from noise [13]. The 6-second period used above corresponds approximately to the duration of the airflow responses seen in the MB (Fig. 2A-C). Resulting ΔF images were divided by the average baseline image to obtain ΔF/F_0_ images.

For studying the spatial distribution of the airflow responses, the ΔF/F_0_ image was averaged over the same 6-second period used for the pixel-by-pixel thresholding described above. For studying airflow responses immediately after airflow on and off in Figure 5A–D, ΔF/F_0_ images were averaged over the 1-second period after airflow on and off respectively. The time courses of the airflow responses for calyces and lobes were calculated by averaging ΔF/F_0_ values of all the pixels that showed significant response to the airflow for each frame.

For the statistical comparison of the airflow on and off responses in the calyx and the lobes, a sliding window (width 1 second) was applied to individual response time courses to determine the maximum response amplitudes during the 3-second period after airflow on (MaxOn) and the 3-seconds period after airflow off (MaxOff). For the statistical comparison of the airflow responses between the control and the antennal-glued flies, each region's ΔF/F values were averaged over the same 6-second period that was used for the calculation of the average response image described above.

For patch analysis, watershed segmentation was applied to the complementary image of the average response image described above. In watershed segmentation, image data is interpreted as a topographic surface where the intensity of the image corresponds to the altitudes, and each segmented region corresponds to individual water catchment basin. To avoid over segmentation, valleys shallower than 20% of the maximum valley depth in each recording were ignored. Airflow-responding pixels in the average response image were grouped according to the region of the watershed segmentation they belonged to, forming “patches” of pixels that represent a spatially connected area sharing a local peak in response to the airflow. Any patches with an area smaller than 1.25 µm^2^ were excluded from the analysis since this is near the resolution of our microscope. The distribution of the patch area for each region of the MB is shown in Figure S6 for *OK107-Gal4; UAS-G-CaMP* flies, and in Figure S9 for *c305a-Gal4; UAS-G-CaMP* and *g0050-Gal4; UAS-G-CaMP* flies. ΔF/F_0_ values for each patch were calculated in the same manner as the ΔF/F_0_ values for different regions of the MB mentioned above, except that the averaging was done within patch rather than across the entire region. Maximum responses after airflow on and off (MaxOn and MaxOff) for each patch were calculated in the same manner as the maximum airflow on and off responses of the calyces and lobes. The original values for the maximum airflow on and off responses were used for the scatter plot of each patch (Fig. 6G–I and Fig. 8F). In some patches, a response to airflow on or off was absent. In a very small proportion of the patches (2 to 6% of the patches in each region), when a response to airflow on or off was absent, the maximum value during this period fell below the baseline. The visual inspection of these responses suggested that this was due to random fluctuations of the fluorescence since they were not time-locked to the stimulus. Because of this, when calculating the On-off Selectivity Index (OSI), these negative values were set to zero to keep the OSI in the range of –1 to 1.

The OSI was calculated for each patch as:
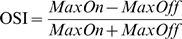
(1)Where the *MaxOn* and *MaxOff* is the maximum airflow on and off responses mentioned above. The OSI ranges from one (only responsive to airflow on) to minus one (only responsive to airflow off).

When quantifying the proportion of the airflow responsive areas that were highly on-selective, highly off-selective, and relatively non-selective, we divided the patches into these three categories according to its OSI. OSIs above 0.2 indicate airflow on responses that are more than 50% greater than the airflow off response, and the patches with these OSIs were categorized as highly on-selective. OSIs below –0.2 indicate airflow off responses that are more than 50% greater than the airflow on response, and the patches with these OSIs were categorized as highly off-selective. Patches with OSIs between 0.2 and –0.2 were categorized as relatively non-selective.

To estimate the anterolateral to posteromedial axis of the α' lobe, we calculated ellipses that had the same second-moments as the shapes of the α' lobes in each recording and used its long axis as an estimate of this axis.

At the end of each experiment, an XYZ scan (z step = 1 µm) covering the entire MB was performed. Basal fluorescence from G-CaMP allowed us to image the structure of MB neurons expressing G-CaMP. These recordings were used to confirm the location of each recording within the entire MB structure and to reconstruct the three-dimensional structure of the MB neurons expressing G-CaMP using Amira (TGS, San Diego, CA).

### Statistics

One-way ANOVA was performed to test the effect of different regions of the MB on maximum responses to airflow on and off, the proportion of pixels responding to the airflow, and the proportion of areas occupied by patches highly selective to airflow on, off, or non-selective. Repeated measures one-way ANOVA was performed to compare the distances among patches that were highly selective to airflow on, off, or non-selective. For all *post hoc* analysis, Tukey HSD correction was used. Unpaired two-tailed Student's *t-test* was used to compare the average response amplitudes and the proportion of the pixels responding to the airflow between control flies and flies with 3^rd^ antennal segment immobilized. It was also used to compare the proportion of patches that were highly selective to airflow off between the α' lobes of *c305a-Gal4; UAS-G-CaMP* and *g0050-Gal4; UAS-G-CaMP* flies. P values for multiple *t-tests* were adjusted using Dunn-Sidak method. Linear regression analysis was used to quantify the relationship between the amplitudes of airflow on and off responses for patches recorded from different regions of the MB. It was also used to quantify the relationship between a patch's OSI and its normalized position along the anterolateral to posteromedial axis of the α' lobe. All statistical analyses were performed using SAS (SAS Institute, Cary, NC).

## Supporting Information

Figure S1 Distributions of recording depths (*OK107-Gal4;UAS-GCaMP*).(A-C) Distributions of recording depths for the calyces (A), the vertical lobes (B), and the horizontal lobes (C) of *OK107-Gal4;UAS-GCaMP* flies. To compare recording depths across flies, the recording depths were normalized in the following manner: for calyces, the top of the calyx was defined as depth 0 and the bottom of the calyx was defined as depth 1. For vertical lobes, the top of the α lobe was defined as depth 0 and the depth where vertical lobes meet horizontal lobes was defined as depth 1. For horizontal lobes, the top of the β' lobe was defined as depth 0 and the bottom of the β lobe was defined as depth 1. Recordings from calyces and horizontal lobes were distributed across all depths while recordings from vertical lobes were concentrated nearer to the tip where the responses were larger. Calyces, n = 33 recordings (from 8 flies), vertical lobes, n = 19 recordings (from 7 flies), and horizontal lobes, n = 16 recordings (from 7 flies).
(0.11 MB TIF)

Figure S2 Principal component analysis of the response pattern. (A) Principal component analysis was performed on all airflow response patterns (ΔF/F_0_ values during the 6-second period after airflow on) recorded from the MB of the OK107-Gal4; UAS-G-CaMP flies (shown in Figure 2) and each response pattern was plotted against its value for the 1st and 2nd principal component (PC). Responses from each region (plotted as circles of different colors) cluster together reflecting their characteristic response pattern. (B) Weight for the 1st and 2nd PC at each time point of the airflow response pattern. The 1st PC corresponds roughly to the overall response amplitude (sign reversed) and the 2nd PC corresponds roughly to the difference between the amplitude of the airflow on and off responses. These two principal components accounted for 66.83 % of the overall variances among the response patterns.
(1.23 MB TIF)

Figure S3 Distributions of recording depths for *c739-Gal4;UAS-GCaMP* and *g0050-Gal4;UAS-GCaMP* flies. (A–B) Distributions of recording depths in α and β lobes of *c739-Gal4;UAS-GCaMP* flies. To compare recording depths across flies, the recording depths were normalized in the following manner: for α lobes, the top of the α lobe was defined as depth 0 and the depth where the α lobe meets the β lobe was defined as depth 1. For β lobes, the top of the β lobe was defined as depth 0 and the bottom of the β lobe was defined as depth 1. As in *OK107-Gal4;UAS-GCaMP* flies, recordings from β lobes were distributed across all depths while recordings from α lobes were concentrated nearer to the tip where the responses were larger. For α lobes, n = 18 recordings (from 7 flies), for β lobes, n = 16 recordings (from 7 flies). (C–D) Distributions of recording depths in α' and β' lobes of *g0050-Gal4;UAS-GCaMP* flies. To compare recording depths across flies, the recording depths were normalized in the following manner: for α' lobes, the top of the α' lobe was defined as depth 0 and the depth where the α' lobe meets the α' lobe was defined as depth 1. For β' lobes, the top of the β' lobe was defined as depth 0 and the bottom of the β' lobe was defined as depth 1. As in *OK107-Gal4;UAS-GCaMP* flies, recordings from β' lobes were distributed across all depths while recordings from α' lobes were concentrated nearer to the tip where the responses were larger. For α' lobes, n = 13 recordings (from 4 flies), for β' lobes, n = 24 recordings (from 6 flies).
(0.12 MB TIF)

Figure S4 Proportion of airflow-responding pixels in the lobes of *c739-Gal4;UAS-GCaMP* and *g0050-Gal4;UAS-GCaMP* flies. (A) Average proportion of the pixels that responded to the airflow stimulation (±s.e.m.) in *c739-Gal4;UAS-GCaMP* and *g0050-Gal4;UAS-GCaMP* flies. Means are significantly different among the different lobes (One-way ANOVA, F_(3,66)_ = 71.81, P<1.0×10^−20^). The α and β lobes had a similar proportion of pixels responding to the airflow stimulation (post hoc Tukey HSD, P>0.05). The α' and β' lobes also had a similar proportion of pixels responding to the airflow stimulation (post hoc Tukey HSD, P>0.05). However, the α' and β' lobes had a much larger proportion of pixels responding to the airflow stimulation compared to the α and β lobes (post hoc Tukey HSD, P<1.0×10^−11^). “*” indicates means that are significantly different at P<1.0×10^−11^. For α lobes, n = 18 recordings (from 7 flies), for β lobes, n = 16 recordings (from 7 flies), for α' lobes, n = 13 recordings (from 4 flies), and for β' lobes, n = 24 recordings (from 6 flies).
(0.09 MB TIF)

Figure S5 Immobilization of the antenna does not affect odor evoked responses in the MB. (A–D) The average amplitude of the response to MCH (A) and BA (C), and the average proportion of pixels responding to MCH (B) and BA (D), in each region of the MB for control flies (solid bars; marked “C”), and flies with glued antenna (bars with diagonal lines; marked “G”) (±s.e.m.). In all regions, immobilization of the antenna did not change the odor-evoked responses (P>0.05). For both MCH and BA, n for the calyces = 7 recordings (from 3 flies), vertical lobes = 9 recordings (from 3 flies), and horizontal lobes = 8 recordings (from 3 flies).
(2.24 MB TIF)

Figure S6 Distributions of the patch area in calyces and lobes of *OK107-Gal4;UAS-GCaMP* flies. (A–E) Histograms showing distributions of the patch areas recorded from calyces (A), α lobes (B), α' lobes (C), β+β' lobes (D), and γ lobes (E). Median areas for the patches in each region are as follows: the calyx = 15.04 µm^2^, the α lobe = 4.33 µm^2^, the α' lobe = 9.37 µm^2^, the β+β' lobes = 14.28 µm^2^, and the γ lobes = 8.61 µm^2^. Bin sizes for histograms are 5 µm^2^ for (A) and (D), 2.5 µm^2^ for (C) and (E), and 2 µm^2^ for (B). n for Calyces, α lobes, α' lobes, β+β' lobes, and γ lobes are the same as in Figures 6G–I.
(0.14 MB TIF)

Figure S7 Comparison of the distribution of the OSIs in different regions of the MB. (A) Empirical cumulative distribution functions for the OSIs of the patches recorded from each region of the MB of *OK107-Gal4;UAS-GCaMP* flies. Cumulative distribution functions show proportions of patches having OSIs under certain values. Distributions of OSIs for the calyx and the α lobe are significantly different from the distributions for the other lobes (P<0.001, two-sided Kolmogorov-Smirnov test, P value adjusted for multiple comparisons using Dunn-Sidak method). The distributions of the OSIs for the other lobes are more skewed towards the lower OSIs (more selective to the airflow off) compared to the distributions for the calyx and the α lobe. Distributions of the OSIs for the calyx and the α lobe are also significantly different from each other (P<0.01, two-sided Kolmogorov-Smirnov test, P value adjusted for multiple comparisons using Dunn-Sidak method). The distribution of the OSIs for the α lobe is more skewed towards lower OSIs (more selective to airflow off) compared to the distribution for the calyx. n for Calyces, α lobes, α' lobes, β+β' lobes, and γ lobes are the same as in Figures 6G–I. (B) Empirical cumulative distribution function for the OSIs of the patches recorded near the tip of α' lobes (between 5 to 15 µm from the tip) in *c305a-Gal4;UAS-GCaMP* and *g0050-Gal4;UAS-GCaMP* flies. Distribution of OSIs for the patches from α' lobes of these two types of flies are significantly different (P = 0.00338, two-sided Kolmogorov-Smirnov test). Number of patches for *c305a-Gal4;UAS-GCaMP* flies = 127 (from 8 recordings in 4 flies), *g0050-Gal4;UAS-GCaMP* flies = 112 (from 8 recordings in 3 flies).
(1.31 MB TIF)

Figure S8 MaxOn and MaxOff are correlated with the anterolateral to posteromedial position in the α' lobe. (A–B) The maximum airflow on (A) and off (B) responses of patches recorded near the tips of α' lobes are plotted against their normalized positions along the anterolateral to posteromedial axis of each section of α' lobes (examples shown by black arrows in Figure 7A–D) (n = 205, 11 recordings from 6 flies). Positions of patches were normalized as in Figure 7F. Maximum airflow on responses are positively correlated with normalized positions of patches (r = 0.298, P = 1.43×10^−5^, n = 205) while maximum airflow off responses are strongly negatively correlated with normalized positions of patches (r = −0.501, P = 2.0×10^−14^, n = 205). Solid black lines indicate linear regression lines. The linear relationships seem to break down at positions close to the anterolateral end of the α' lobe.
(0.13 MB TIF)

Figure S9 Distributions of the patch area for the α' lobes of *c305a-Gal4;UAS-GCaMP* and *g0050-Gal4;UAS-GCaMP* flies. (A–B) Histograms showing distributions of the patch areas in α' lobes of *c305a-Gal4;UAS-GCaMP* flies (A) and *g0050-Gal4;UAS-GCaMP* flies (B). Only the patches from recordings made between 5 to 15 µm from the tips of the α' lobes are included. Median area for the patches was 8.65 µm^2^ in *c305a-Gal4;UAS-GCaMP* flies and 19.32 µm^2^ in *g0050-Gal4;UAS-GCaMP* flies. Bin sizes for histograms are 2 µm^2^ for (A), and 3 µm^2^ for (B). n are the same as in Figure S7B.
(0.13 MB TIF)
