# Supplementary figures and images for: Neural Representations of Airflow in Drosophila Mushroom Body
Source: PLoS One. 2008 Dec 30;3(12):e4063. doi: 10.1371/journal.pone.0004063 (PMC2603598; doi:10.1371/journal.pone.0004063)

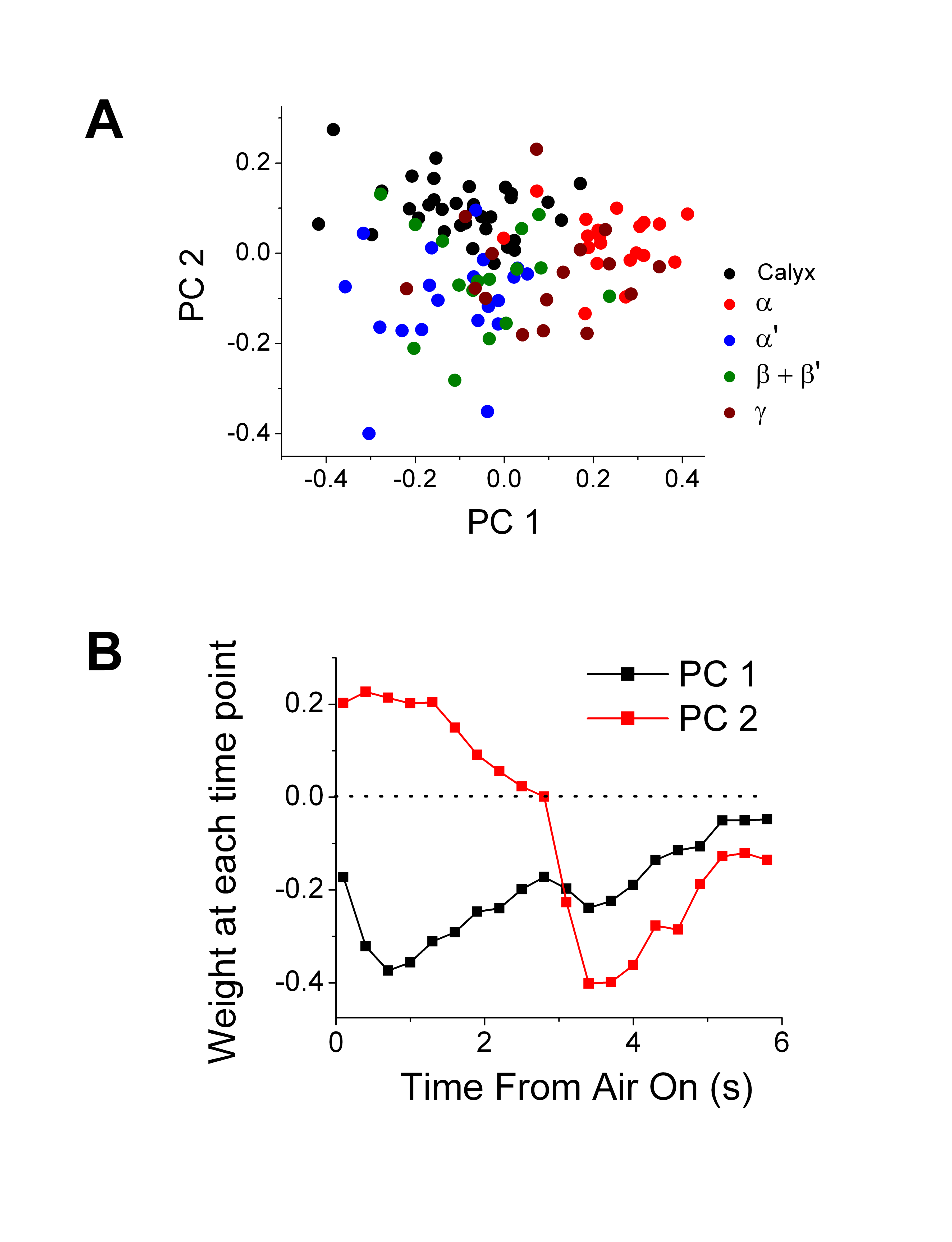

Supplement: Figure S2 — Principal component analysis of the response pattern. (A) Principal component analysis was performed on all airflow response patterns (ΔF/F0 values during the 6-second period after airflow on) recorded from the MB of the OK107-Gal4; UAS-G-CaMP flies (shown in Figure 2) and each response pattern was plotted against its value for the 1st and 2nd principal component (PC). Responses from each region (plotted as circles of different colors) cluster together reflecting their characteristic response pattern. (B) Weight for the 1st and 2nd PC at each time point of the airflow response pattern. The 1st PC corresponds roughly to the overall response amplitude (sign reversed) and the 2nd PC corresponds roughly to the difference between the amplitude of the airflow on and off responses. These two principal components accounted for 66.83 % of the overall variances among the response patterns. (1.23 MB TIF) [file pone.0004063.s002.tif]

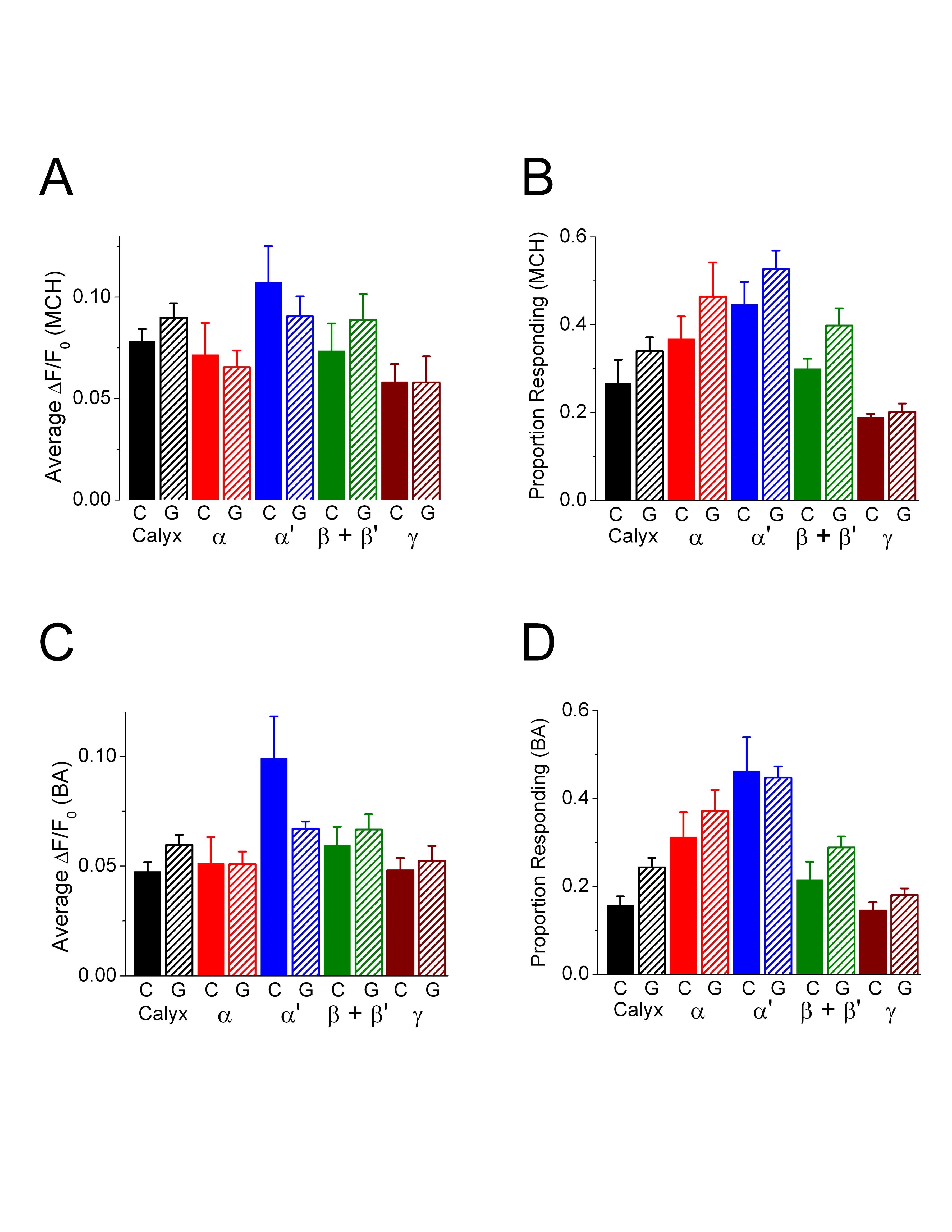

Supplement: Figure S5 — Immobilization of the antenna does not affect odor evoked responses in the MB. (A–D) The average amplitude of the response to MCH (A) and BA (C), and the average proportion of pixels responding to MCH (B) and BA (D), in each region of the MB for control flies (solid bars; marked “C”), and flies with glued antenna (bars with diagonal lines; marked “G”) (±s.e.m.). In all regions, immobilization of the antenna did not change the odor-evoked responses (P>0.05). For both MCH and BA, n for the calyces = 7 recordings (from 3 flies), vertical lobes = 9 recordings (from 3 flies), and horizontal lobes = 8 recordings (from 3 flies). (2.24 MB TIF) [file pone.0004063.s005.tif]

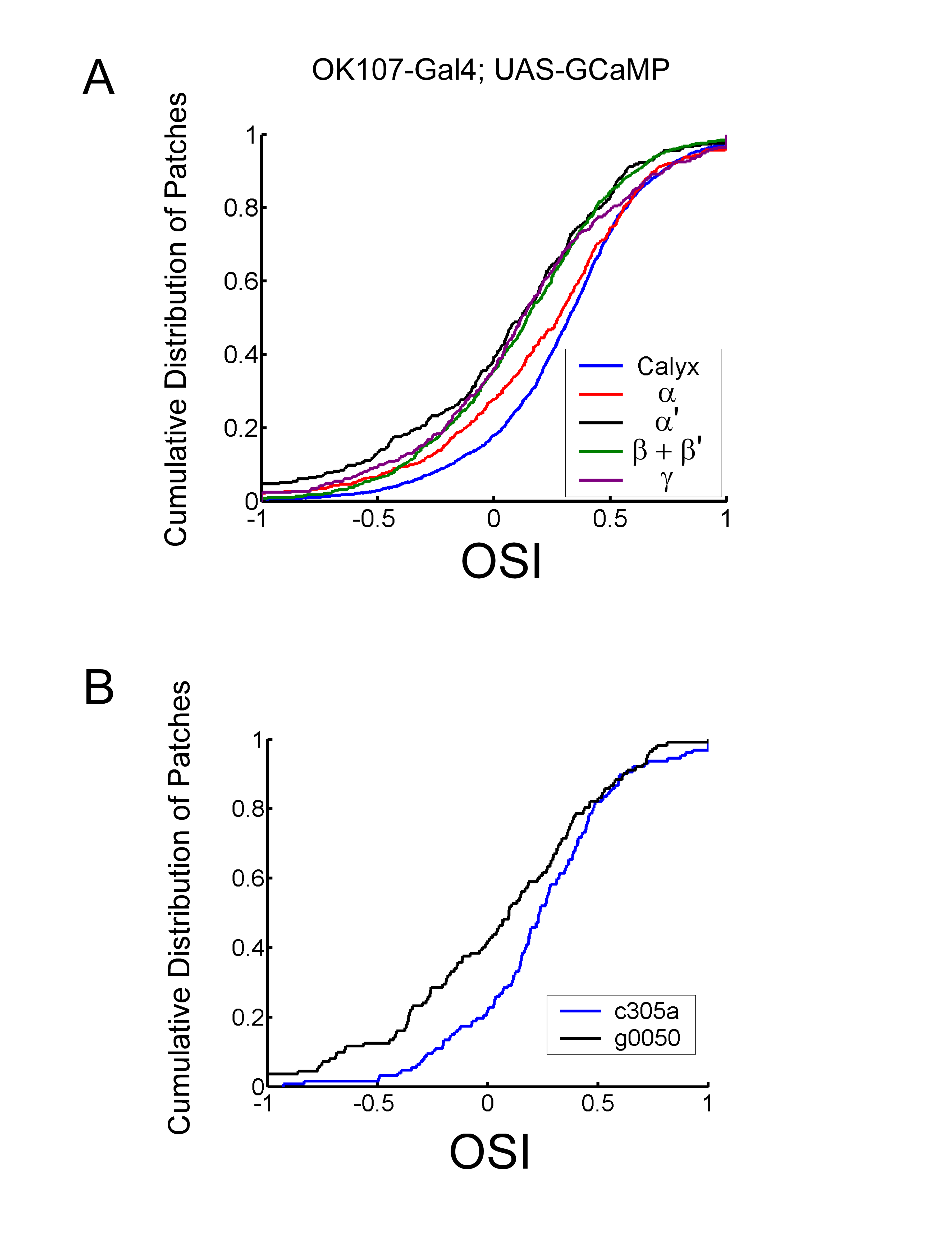

Supplement: Figure S7 — Comparison of the distribution of the OSIs in different regions of the MB. (A) Empirical cumulative distribution functions for the OSIs of the patches recorded from each region of the MB of OK107-Gal4;UAS-GCaMP flies. Cumulative distribution functions show proportions of patches having OSIs under certain values. Distributions of OSIs for the calyx and the α lobe are significantly different from the distributions for the other lobes (P<0.001, two-sided Kolmogorov-Smirnov test, P value adjusted for multiple comparisons using Dunn-Sidak method). The distributions of the OSIs for the other lobes are more skewed towards the lower OSIs (more selective to the airflow off) compared to the distributions for the calyx and the α lobe. Distributions of the OSIs for the calyx and the α lobe are also significantly different from each other (P<0.01, two-sided Kolmogorov-Smirnov test, P value adjusted for multiple comparisons using Dunn-Sidak method). The distribution of the OSIs for the α lobe is more skewed towards lower OSIs (more selective to airflow off) compared to the distribution for the calyx. n for Calyces, α lobes, α' lobes, β+β' lobes, and γ lobes are the same as in Figures 6G–I. (B) Empirical cumulative distribution function for the OSIs of the patches recorded near the tip of α' lobes (between 5 to 15 µm from the tip) in c305a-Gal4;UAS-GCaMP and g0050-Gal4;UAS-GCaMP flies. Distribution of OSIs for the patches from α' lobes of these two types of flies are significantly different (P = 0.00338, two-sided Kolmogorov-Smirnov test). Number of patches for c305a-Gal4;UAS-GCaMP flies = 127 (from 8 recordings in 4 flies), g0050-Gal4;UAS-GCaMP flies = 112 (from 8 recordings in 3 flies). (1.31 MB TIF) [file pone.0004063.s007.tif]
